# Does organ donation legislation affect individuals' willingness to donate their own or their relative's organs? Evidence from European Union survey data

**DOI:** 10.1186/1472-6963-8-48

**Published:** 2008-02-27

**Authors:** Elias Mossialos, Joan Costa-Font, Caroline Rudisill

**Affiliations:** 1 LSE Health, London School of Economics, London, UK; 2 European Institute, London School of Economics, London, UK

## Abstract

**Background:**

Maintaining adequately high organ donation rates proves essential to offering patients all appropriate and available treatment options. However, the act of donation is in itself an individual decision that requires a depth of understanding that interacts with the social setting and the institutional framework into which an individual is embedded. This study contributes to understanding factors driving organ donation rates by examining how country regulation, individuals' awareness of regulatory setting, social interactions and socio-demographic determinants influence individuals' willingness to donate their own organs or those of a relative.

**Methods:**

We draw representative data from the Eurobarometer survey 58.2 undertaken in 2002 with respondents throughout the European Union to capture heterogeneity in institutional setting. We use logistic regression techniques to estimate the determinants of willingness to donate one's own organs and those of a deceased relative. We employ interaction terms to examine the relationship between institutional setting and respondent's awareness of organ donation legislation in their country.

**Results:**

Our findings indicate that individuals are more likely to donate their organs than to consent to the donation of a relative's organs. Both decisions are affected by regulation (presumed consent), awareness of regulation and social interactions such as the ability to count on others in case of a serious problem (reciprocity). Furthermore, education (more educated), age (younger), expressing some sort of political affiliation determine willingness to donate one's own organs and consent to the donation of those of a relative.

**Conclusion:**

This study confirms and develops further previous research findings that presumed consent organ donation policy positively affects the willingness of individuals to donate their own organs and those of relative by highlighting the importance of awareness of this regulation and an individual's level of social interactions in making choices about donation. Results found using interaction terms underline the importance of population awareness of organ donation legislation as well as the legislation type itself. Findings also point to the role of social interactions in influencing individuals' willingness to donate their organs or those of a relative.

## Background

Although progress in medical science and technology has vastly improved success rates for organ transplantations, severe organ shortages continue preventing these medical advances from being realized for all potential patients. Efforts to expand the available organ supply have become more crucial for meeting transplant demand. However, often available organ transplants might well depend on institutional frameworks rather than on individual demand, namely the specific regulations in each country as well as individuals' awareness of this legislation.

In June 2006, the European Commission issued a consultation document concerning the state of organ donation and transplant policy at the European level [1]. The discussion of European Union (EU) level policy regarding organ transplant and donation policy highlights not only the heterogeneity of organ donation policy throughout Europe but also the importance of understanding what successful policies could be enacted. A clearer picture of the decision-making process behind organ donation rates should inform this policy process. While some analysis has been undertaken on the empirical determinants of effective organ procurement rates, evidence on the underlying behavioral explanations of such decisions and the extent to which they are influenced by the particular legislative setting to which individuals are subject has been more limited [2].

This paper contributes to the literature by examining how individuals' perceptions about institutional frameworks affect individuals' willingness to donate their own organs and those of relatives by using data from the European Union and extending findings of other studies attempting to compare European Union experience with that of the US [3]. We examine the influence of national-level organ donation policy (presumed consent, enforced presumed consent and informed consent) as well as awareness of this legislation along with other individual determinants of willingness to become a posthumous organ donor and consent to the donation of a deceased family member's organs. We use an interactive variable to specifically look at how the role of legislation type and awareness of that legislation might depend on each other to impact willingness to donate. We also examine the roles of social interactions (and collective efficiency), having a political affiliation (indication of interest in community affairs) and specific political affiliation in determining willingness to donate. Understanding the role of the community allows us to examine whether choosing to donate has underlying societal motivations since donation might be seen as an implicit communal contract with others. Furthermore, we control for country specific effects so that correlated effects impacting individuals' values would be captured [4]. Finally, we examine the common influence of socio-economic (education) and demographic characteristics (age, gender) that could act as individuals' incentives to donate or to consent the donation of a relative's organ.

### Policy approaches and institutional background

A lack of organs to meet present demand has resulted in long transplantation waiting lists in the US and across Europe. In Western Europe, nearly 40,000 patients were waiting for a kidney transplant in 2003 [5]. In the US, demand for organs overstretches supply partly because only 42 percent of eligible organ donors end up actually donating [6].

Governments put forward a variety of policy approaches to improve organ transplant waitlists. European countries are classified according to two types of institutional settings for confronting organ transplant needs: informed consent (opt-in) or presumed consent (opt-out). In countries with informed consent or 'opt-in' legislation, such as the UK, Germany, and Sweden, an individual or his/her family must give explicit permission for organ removal. Presumed consent countries such as Spain, Portugal, and Austria, assume universal consent without explicit registration otherwise. The latter is more prominent in the EU although countries with presumed consent legislation can differ in enforcement levels. Enforced presumed consent policy means that individuals who have not opted out of organ donation will automatically donate their organs upon time of death if organs are in a suitable clinical condition. Otherwise, unenforced presumed consent policies have caveats such as allowing relatives to refuse their relative's organ donation even though the relative has not explicitly opted-out. Procurement data across 22 countries worldwide indicates that presumed consent policy has an impact on donation rates [2]. Gimbel, Strosberg, Lehrman, Gefenas & Taft and Johnson and Goldstein also found that presumed consent policy increases organ donations in Europe [7,8].

In practice, most families are consulted with regard to a deceased relative's organ donation even if this relative has already expressed willingness to be a donor. Approximately half of families in the US and 42 percent of families in the UK refuse requests for the donation of a relative's organ in comparison to 20 percent objecting to donation in Spain [9-11]. Compared to an individual's decision to donate their own organs, there are limited studies examining the determinants of willingness to consent to the donation of a deceased family member's organs. Just as Bowles finds that institutional setting prompts individuals to display certain preferences, we expect that organ donation policy will have an impact on individuals' propensities to donate their own organs and those of others [12].

While organ donation policies might impact donor rates, individual attitudes regarding donations are not always aligned with donation behavior. Though European surveys show widespread public support for organ donation, donor card signing (opt-in) does not reflect this opinion. In Germany, only 7–10 percent of individuals who are in favor of organ donation carry a donor card [13]. Only 19 percent of all adults in the UK have registered on the national donor registry [10].

However, an individual's willingness to donate (WTD) is an expression of the intention to pursue a behavior; therefore WTD provides a better indication of donation rates in a country than simply support of organ donation. Individuals can express favor towards organ donation as an abstract concept for society to encourage but they may not be reflecting how they feel about donation for themselves or a family member.

### Role of individuals' characteristics in decision-making

Apart from the impact of the institutional setting on organ donation, previous studies have shown that individual characteristics such as age, gender, education level, income level and religious associations can play a significant role in determining likelihood of an individual's organ donation and/or consent to a relative's donation. Studies on how age affects organ donation show that older people are less likely to donate [14,15] while middle aged individuals [16] are slightly more willing to donate.

Most studies find relatively little difference between genders in willingness to donate [16] while some find that females are more likely to donate [15]. Differences between willingness to donate and actual donation practice could have many explanatory factors including cause of death and family decision-making at the time of death potentially differing based upon gender. For example, in the US, families of white, young male patients were more likely to consent to donation than families of other types of patients [17].

Of the many socio-economic factors that have been associated with willingness to donate organs, education level is consistently one of the most explanatory. Education might stand as a proxy for knowledge about health related issues. Studies in the U.S. estimate that individuals who have attended graduate or professional school are approximately 1.5 to 3 times more likely to be willing to become a posthumous donor than individuals who have solely completed high school [15,16].

In the UK, more affluent individuals are less likely to donate [18] while higher income is associated with higher willingness to donate in Canada [19]. Siminoff, Gordon & Hewlett et al.'s study of family consent for the donation of a relative's organ found no association between consent rates and families' educational attainment or income [17]. However, knowing that a family member wanted to donate his/her organs has been found to be strongly associated with familial consent [17,20,21].

Knowledge about organ donation policy and the organ donation process have also been found to increase individuals' willingness to donate their own organs and those of a relative [22]. Fear can drive negative outcome expectations, which have been shown to be negatively related to likelihood of registering to be a donor [23]. Increased knowledge could alleviate this anxiety connected to being an organ donor through disseminating factual information to counteract fears [24].

With the heterogeneity of policy and social preferences in the EU, studying the variety of European experience with organ donation permits an assessment of the implications of organ donation policies.

### The influence of social interactions and collective efficiency

The willingness to donate one's organs can be viewed as an expression of an individual's reciprocity, namely a mechanism by which an individual pays-back society for inclusion and social support they have experienced and hope to experience in the future. Accordingly, one might expect individuals with a higher sense of inclusion and those more involved in social interactions to be more willing to donate their organs. One might wish to distinguish between contextual interaction*s*, resulting from group interaction and group composition and pure correlation effects, created by a single shared similar characteristic of a group. As noted in Manski, the problem lies in distinguishing between these two effects [4]. Contextual effects can be controlled for, for example, by using geographical variables while the latter can be measured by asking individuals whether they could count on others and whether they interact with people in their social setting (e.g., neighbors). This study examines the correlation effect of inclusion in social setting (relationships with neighbors) to identify any impact of collective efficiency on organ donation opinions. Furthermore, these variables would measure the quality of interactions, which are clearly different from specific characteristics of individuals (e.g., household size) for which we might control. In this case we suggest, that if individuals share the single characteristic of feeling socially including through social interactions then these individuals would be more likely to be willing to then give back to this society they feel a part of through organ donation.

## Methods

We use Eurobarometer survey 58.2, a cross-national comparative survey that includes a representative national sample of 15 EU countries (Austria, Belgium, Denmark, Finland, France, Greece, Ireland, Italy, Luxembourg, Netherlands, Portugal, Spain, Sweden, Germany, United Kingdom). Data were collected over the period October 2002 – November 2002 but the survey was released in January 2004. The Eurobarometer survey series is designed to regularly monitor social and political attitudes of the EU public. This survey was performed under the ethical guidelines defined by the Public Opinion Analysis Sector of the Directorate General Press and Communication of the European Commission. The sample universe includes resident citizens of the EU aged 15 and over with a total number of 16,230 survey respondents. The survey was conducted on a multi-stage random sampling basis so that the total survey sample is representative of the whole territory of the 15 countries surveyed. The first stage of random sampling occurred on the national level according to each country's distribution of metropolitan, urban and rural residents. In the second stage, a cluster of addresses was randomly selected from each primary sampling unit. Addresses were chosen systematically using standard random route procedures, beginning with an initial address selected at random. Respondents within each household were selected at random for face-to-face interviews. Caveats to the survey include sampling procedure methods and difficulties associated with measuring income and education among EU member states [25-27].

This study uses logistic regression to analyze the determinants of individuals' willingness to become cadaveric organ donors and to permit the donation of a relative's organs, which are the two outcomes of interest. The 4-point Likert scale used to elicit responses about organ donation fits this kind of model because responses are not ordinal in nature. The question, 'whatever the rules and regulation, would you personally be prepared to donate one of your organs to an organ donor service immediately after your death' had the response options of '1' meaning 'yes, definitely' to '4' meaning 'no, definitely, not' and then '5' meaning 'don't know.' The question, 'in hospital, if you were asked, would you give your consent to the donation of an organ from a deceased relative' had three responses of 'yes,' 'no,' and 'don't know.' When running the multivariate regression, we treated the "don't know" responses for organ donation or donation of a deceased relative's organs as "no" responses (0 s) in a conservative method to maintain the number of observations and because both express a failure to be positively certain about donating organs. We tested for sample selection when necessary but the inverse mills lambda was never significant thus suggesting that excluding the 'don't know' responses from the questionnaire exerts no influence on the results. We estimated using logistic models to compare across the two outcomes of interest namely: willingness to donate own organs and relative's organs. We clustered our data by country. The clustering method used meant that respondents were grouped according their country of residence thus accounting for country specific effects and treating clusters rather than observations as independent [28]. In the same fashion as in multilevel modeling, we have overcome some deficiencies in existing datasets such as the bias caused by clustering on a country basis.

Both models include variables for self-perceived presence of serious illness (yes, no), self-perceived level of health (very bad, bad, good), gender, political affiliation (left, center, right, none), age, urban or rural place of living, formal education (finished education at 15 or below, 16–19 or 20+, still studying), awareness of legislation type in their country (yes, no), type of organ donation legislation (unenforced presumed consent, enforced presumed consent, informed consent), interviewee cooperation (excellent, fair, average, bad), number of people can count on in case of serious problems (none, 1 or 2, 3–5, 5+) and ease of access to help from neighbors (very easy, easy, difficult of very difficult). (see Table A1 in Additional file 1 for explanatory variable definitions and descriptive statistics). We created variables to group countries by whether they have unenforced presumed consent, enforced presumed consent or informed consent policies to address if country institutional setting (legislation) impacts individuals' attitudes about organ donation. The variable about the number of people respondents can count on in case of problems measures sense of social inclusion while the variable capturing help from neighbors measures social interactions. Both of these variables are considered indicators of social support level and collective efficiency allowing us to analyze correlation effects of social setting. We use interactive variables to further investigate the impacts of legislation type and awareness of legislation by examining the interaction of these two variables together on willingness to donate. This allows us to disentangle a caveat from previous studies, namely that for the institutional setting to effectively determine behavior it must be perceived as constraining individuals' decisions.

## Results

### Descriptive statistics

Of the sample of Europeans questioned, 60.1 percent are willing to be organ donors while only 48.4 percent are willing to consent to the donation of a relative's organs. 31.9 percent of respondents were from countries with unenforced presumed consent, 28.7 percent from countries with enforced presumed consent and 39.0 percent from countries with informed consent policies. Only 31.0 percent of Europeans expressed awareness of their country's type of organ donation legislation.

Table 1 provides evidence indicating that institutional setting matters given that regulation type appears to be an important factor in individuals' willingness to donate. As a general pattern, we find that informed consent countries' respondents exhibit a lower probability of willingness to donate and to consent to the donation of a relative's organs. This can be taken as preliminary evidence that attitudes are shaped by the institutional setting in which individuals are a part thus supporting the finding reached by Bowles [12]. However, one might question this result given that other factors might be impacting attitudes such as differences in how individuals interact [4] or in the efficiency in pursuing collective action as a society. In addition, it is important to keep in mind, that policy choices can also be shaped by the culture of a given country and vice versa, regulations also might change people's attitudes.

**Table 1:** Willingness to donate one's own and consent to donating a relative's organs by institutional setting (regulation)

	**Willingness to donate own organs**			**Willingness to donate relative's organs**		
	N	Mean	s.e	N	Mean	s.e

Total	16230	0.60	0.004	16230	0.48	0.004
Presumed consent	5174	0.64	0.007	5174	0.51	0.007
Presumed consent enforced	4654	0.60	0.007	4654	0.51	0.007
Informed consent	6402	0.57	0.006	6402	0.44	0.006

Of the population sample studied, 26.3 percent reported a major illness while only 6.0 percent declared themselves to be of bad or very bad health. This result could reflect respondents adapting to their state of illness and not perceiving their major illness to be as bad as a healthy person would think it is. About half of the sample was male and 23.1 percent declared no political affiliation. 37.1 percent of the sample live in rural areas and 25.9 percent finished education by age 15 or below. Only 1.2 percent of the sample exhibited poor cooperation with the interviewer. About 3.6 percent of the sample could not count on someone else in case of a serious problem. 34.1 percent could count on 1 or 2 people, 35.0 percent could count on 3 to 5 people and 24.9 percent could count on more than 5 people. About 73.0 percent expressed the ability to access help from neighbors with 26.8 describing this help as very easy to access and 46.2 percent describing it as easy to access. 6.3 percent found it difficult to get help from neighbors and 6.5 percent found it very difficult.

The proportion of respondents who described themselves as definitely or likely willing to be an organ donor differed significantly between countries. For example, less than one half (49%) of Germans expressed willingness to donate versus three-quarters (75%) of all Swedes. The percentages of national populations expressing likely or absolute refusal to donate varied greatly from 14 percent of the Spanish surveyed to 32 percent of the Germans. There was also wide variation in the proportion of individuals who were not sure if they would be willing to donate. 10 percent of Swedes (the least indecisive) expressed indecisiveness while Spanish respondents expressed the most indecisiveness at 25 percent of all respondents.

Respondents from different countries expressed variation in their willingness to consent to the donation of a relative's organ in hospital. Germans were the least likely to consent to the donation of a relatives' organ at 36 percent of respondents while Swedes were the most likely at 64 percent. Overall, respondents were much more likely to be unsure about consenting to the donation of an organ from a deceased relative. For the EU as a whole, 30 percent of the population responded "Don't Know" when asked if they would consent to the donation of a deceased relative while 17 percent offered the same response when making a decision about their own donor status. 37 percent of respondents from Spain were indecisive regarding donation of a relative's organ versus 25 percent when asked the same question about themselves. Respondents from Italy and the UK followed closely behind those from Spain with 36 percent being indecisive about consenting to the donation of a relative's organs. As was the case in being asked about their own willingness to donate, Swedish respondents were the least indecisive in making decisions about their relative's donation of organs.

82 percent of people who said they would 'definitely' be willing to donate their own organ also said they would be willing to consent to the donation of one from a deceased relative. Likewise, 76 percent of people who said they would 'definitely not' donate their own organ also said they would not consent to the donation of one from a deceased relative.

### Multivariate regression analysis

Regression results further develop the findings of these descriptive statistics. The diagnostic tests of Pseudo R^2 ^and percentage of correctly predicted responses indicate that the models predict a significant share of the expected cases, and the log likelihood test rejects the hypothesis of all variables being equal to zero. Values for these tests are found in Table 2. Furthermore, we tested for the existence of multicollinearity as a result of the interactions and found that because the variance inflation factors (VIF) were systematically below five, multicollinearity is not an issue.

**Table 2:** Determinants of Willingness to Become a Donor and Consent to the Donation of a Deceased Relative's Organ in Hospital

	**Willingness to donate own organs**				**Willingness to donate relative's organs**			
	Odds ratio	t-value	+95% C.I.	-95% C.I.	Odds ratio	t-value	+95% C.I.	-95% C.I.

*Needs and Related Socio-Demographics*								

*No illness*								
Illness	1.10^a^	3.48	*1.20*	*1.00*	1.22^a^	5.19	*1.32*	*1.12*
*Good health*								
Very bad health	0.84	-0.91	1.15	0.53	0.85	-0.81	1.16	0.54
Bad health	0.86	1.85	1.00	0.72	0.77^a^	-3.17	*0.89*	*0.65*
*Female*								
Male	1.02	0.47	1.08	0.96	1.05	1.55	1.11	0.99
*Age over 60*								
Age under 30	1.57^a^	5.94	*1.81*	*1.33*	1.32^a^	3.74	*1.52*	*1.12*
Age 30 to 45	1.58^a^	9.45	*1.74*	*1.42*	1.34^a^	6.27	*1.46*	*1.22*
Age 45 to 60	1.34^a^	6.37	*1.46*	*1.22*	1.22^a^	4.43	*1.34*	*1.10*

*Political Affiliation*								

*Don't know political identification*								
Left politics	1.73^a^	10.92	*1.91*	*1.55*	1.62^a^	9.90	*1.78*	*1.46*
Center politics	1.51^a^	9.04	*1.65*	*1.37*	1.51^a^	9.16	*1.65*	*1.37*
Right politics	1.42^a^	6.65	*1.58*	*1.26*	1.56^a^	8.49	*1.72*	*1.40*

*Social Interactions*								

*Support from no one*								
Support from 1 to 2 others	1.28^a^	3.32	*1.46*	*1.10*	1.36^a^	4.12	*1.56*	*1.16*
Support from 3 to 5 others	1.43^a^	4.84	*1.65*	*1.21*	1.45^a^	4.87	*1.67*	*1.23*
Support from over 5 others	1.50^a^	5.21	*1.74*	*1.26*	1.60^a^	6.00	*1.85*	*1.35*
*Very difficult or difficult to receive help from neighbors*								
Very easy to receive help from neighbors	1.32^a^	5.74	*1.44*	*1.20*	1.32^a^	5.92	*1.44*	*1.20*
Easy to receive help from neighbors	1.11^b^	2.55	*1.21*	*1.01*	1.13^a^	2.97	*1.23*	*1.03*

*Socio-Economics*								

*Urban*								
Rural	1.00	0.11	1.08	0.92	1.06	1.67	1.14	0.98
*Still studying*								
Stopped education at 15 years	0.63^a^	-5.40	*0.73*	*0.53*	0.69^a^	-4.48	*0.81*	*0.57*
Stopped education at between 16–19 years	0.78^a^	-3.13	*0.90*	*0.66*	0.86^b^	-1.99	*0.98*	*0.74*
Stopped education at over 20 years	1.00	0.03	1.16	0.84	1.07	0.83	1.23	0.91

*Institutional Setting and Knowledge*								

*Unaware*								
Awareness	1.91^a^	11.01	*2.13*	*1.69*	1.74^a^	9.85	*1.94*	*1.54*
*Informed consent*								
Presumed consent	1.17^a^	3.33	*1.29*	*1.05*	1.27^a^	4.95	*1.39*	*1.15*
Presumed consent enforced	1.29^a^	5.21	*1.41*	*1.17*	1.56^a^	9.24	*1.72*	*1.40*
*Interaction between awareness and informed consent*								
Interaction between awareness and presumed consent	1.18	1.69	1.40	0.96	1.05	0.54	1.23	0.87
Interaction between awareness and presumed consent enforced	1.48^a^	4.34	*1.73*	*1.23*	1.20^b^	2.17	*1.40*	*1.00*

*Control Variables*								

*Bad cooperation*								
Excellent cooperation	1.78^a^	3.80	*2.31*	*1.25*	1.88^a^	3.92	*2.47*	*1.29*
Fair cooperation	1.23	1.39	1.60	0.86	1.30	1.64	1.71	0.89
Average cooperation	0.97	-0.18	1.26	0.68	1.03	0.17	1.36	0.70

Pseudo R^2^	0.084				0.054			
Adjusted LR chi2(22)	967.22				840.61			
Log likelihood	-10430.4				-10821.6			
% correctly predicted responses	73.30%				78.77%			

Note: C.I. stands for confidence interval

Baseline category appears in italics

^a ^Significant at 1% ^b ^Significant at 5%

Table 2 provides evidence of the positive relationship between educational attainment and awareness of the institutional setting (legislation) on the willingness to become an organ donor after death and consenting to the donation of a relative's organ. Those who had finished their studies by the age of 15 were approximately half as willing to become a cadaveric donor as those who had completed additional schooling. Even those who had finished their schooling between the ages of 16–19 were considerably less likely to donate than those who had studied past the age of 20.

Examining the demographic variables and consistently with prior studies, gender appears to have a negligible influence on willingness to donate while age exerts greater influence. Older individuals (60+ years) were considerably less willing than other age groups to become posthumous organ donors. Again, this finding is consistent with previous findings regarding age and willingness to donate [14,15]. Willingness to donate was the highest for individuals less than 45 years old. The propensity for younger individuals to consent to organ donation might be due to less thought being given towards death at a young age because it seems farther away than for older respondents.

Living in a rural area and type of political affiliation have no significant effect on willingness to donate but having some political affiliation (as compared as not revealing any political affiliation) is significantly associated with willingness to donate.

We use two variables (number of people respondent can count on and difficulty in getting help from neighbors) to examine the particular influence of social interactions. We find that individuals who report having fewer people they could really count on were less likely to become organ donors after their death*s*. Having more than two people to count on appears to increase the odds of an individual donating their organs by 43 to 50 percent and a relative's organs by 45 to 60 percent. Similarly, although not exhibiting a linear effect, individuals reporting more difficulty in getting help from their neighbors were less likely to be willing to donate.

We also examine the effects of regulation, the awareness of regulation and the interaction between both variables. This analysis found that countries with a presumed consent policy had respondents with a higher willingness to donate their own organs as well as those of a relative. The result is even stronger in countries enforcing a presumed consent policy. This may indicate that organ donation policy might be endogenous, meaning that it reflects prior public attitudes and values. Awareness of regulation increases the odds of being willing to donate one's own organs by 91 percent and those of a relative by 74 percent.

Finally, the interaction variable of enforced presumed consent policy and policy awareness was significant in impacting individuals' willingness to donate. This interaction variable was significant at the 99% level whereas the interaction between non-enforced presumed consent and awareness of this policy was significant at the 90% level. This indicates that in countries where presumed consent is enforced, awareness exerts a specific non-linear effect on willingness to donate.

## Discussion

This study has explored evidence of willingness to donate using data representative of the European Union. We find that institutional setting and awareness of institutional setting affect not only procurement rates as some studies suggest, but also the willingness of people to donate their organs. On the other hand, people seem to be more willing to donate their own organs, as they might feel more prepared to make that decision, than those of their relatives, even after controlling for a set of relevant controls.

One major contribution of this study appears in the finding of a significant association between willingness to donate and how people view their level and strength of social support both in terms of how many people they can rely on in case of a serious problem and how difficult it is to get help from neighbors. This evidence suggests that the decision to donate one's own organs or those of a relative could have a relationship with the extent to which individuals receive frequent support from others. This finding highlights the importance of how a sense of inclusion may influence individuals' feelings of reciprocity with the society in which they live. These results in no way imply causality but a higher likelihood of individuals with greater social interactions to support donation of their own organs as well as those of others.

We have found that individuals' responses regarding organ donation for themselves often translated into the same opinion about the donation of a deceased relative's organs, however respondents appear much more unsure regarding decisions about a relative's organ donation than when making decisions about themselves. While an individual making his own choice about organ donation increases the likelihood of donation, thinking about becoming an organ donor implies contemplating one's own death. Some individuals might be reluctant to decide about becoming an organ donor since it requires thinking about an event they would rather prevent and not acknowledge its possibility of occurrence.

As for the control variables, our results support previous studies in showing a clear association between willingness to donate and level of formal education and age, and a negligible association between willingness to donate and gender. Younger respondents who have more years of education and are more aware of their country's type of donation legislation tend to be more willing to donate their own organs as well as consent to the donation of a relative's [14,15]. We also find some evidence of long-standing illness explaining donations as individuals who find themselves increasingly likely to need an organ more intensively perceive the benefits of organ donation. However, bad or very bad health status is not associated with an individual's willingness to donate. Gender, being an urban or rural resident and type of political affiliation appear not to be determinants of willingness to donate, however, having some political affiliation increases the likelihood of willingness to donate [16]. The explanatory power of having a political affiliation might be due to the fact that those individuals revealing some political affiliation could be more concerned with the feeling of being involved in the collective organization of society and interactions with others. These activities could be less important to someone without a political affiliation thus leading them not to become an organ donor. Among those stating their affiliation, those with views left of center seem to be more likely to donate than those at the center and right of center.

This study shows that decision making about organ donation by relatives of the deceased rather than the potential donor prior to death may have a downward impact on organ supply. This result is consistent with Johnson and Goldstein's finding that family objections to a love one's consent might play a role in determining actual donation rates [8]. Reluctance on the part of relatives is not surprising however, because of the emotional factors incorporated into making decisions for someone else posthumously with perhaps little or no insight into that individual's wishes. Decisions made about donating a relative's organs are often made under a quick and stressful situation where the default 'no' position seems safer [29].

Furthermore, we have found that countries with presumed consent regulation have a higher willingness to donate, especially if this policy is enforced. This finding supports previous research showing that presumed consent legislation increases organ procurement levels [2,7].

The particular arguments supporting presumed consent policy revolve around the fact that making a decision on donating organs might require some effort (e.g., filling out a form) while reacting to or accepting the regulation might be effortless [8]. Therefore, presumed consent policy lends itself towards higher procurement levels because of individuals' tendencies to fail in performing the active decision-making efforts required with opt-in organ donation legislation. Given that individuals might not experience utility from thinking about death, one might argue that where organ donation policy is solely based upon informed consent, the state of choosing not to donate is likely to prevail. Therefore, the reason procurement rates might tend to be lower in countries with informed consent legislation could be that individuals tend to not make a decision and therefore do not end up donating organs.

Our results indicate that individuals' awareness of the legislation has a significant effect on willingness to donate, indicating that efforts to improve educational programs and informational campaigns on the social and health benefits of organ donation could contribute to increase the number of donations. This result contrasts that of Beard, Kaserman and Saba's study where education programs were not found helpful in impacting organ donation rates [30] but supports findings that increases in knowledge about organ donation can positively influence likelihood of registering for organ donation [22,24].

An interaction variable between regulation and awareness of the regulation appears as a strong and significant determinant of willingness to donate. This interaction variable tests the determinative power of not only regulation but also whether respondents are aware of this regulation. Findings suggest that not only does type of regulation but also awareness of regulation determine willingness to donate as well as donate organs of a relative.

Analysis performed in this study met several limitations that apart from deserving mention, could also guide future research. In general, the percentages of the variance (R^2^) explained by the models presented in this study are 8.4% and 5.4%, suggesting that other variables not included in this study could also have a significant effect on individuals' willingness to donate their own organs and those of others. By the authors of this study not being involved in setting the specific questions to be asked by the Eurobarometer survey, the survey's design limited this study. In particular, the data lacks information about how religious beliefs and ethnicity impact decisions around organ donation. Additionally, the survey does not include data on family structure apart from marital status such as how many individuals live in the respondents' household, which could prove important when looking at relatives' willingness to donate organs since those without close relatives might look upon such decisions unfavorably. Other potential determinants of willingness to donate, which this study could not address because of data limitations were risk perceptions about donation procedures or differing treatment at hospital based upon donation status, knowledge about a relative's wishes for organ donation influencing a relative's choices, and to add to previous work on the role of information, if greater knowledge about the donation process would improve willingness to donate in any institutional setting. A further issue related to the data is potential endogeneity in the policy awareness variable. Individuals being aware of their country's organ donation policy could be endogenous to willingness to donate as those who are willing to donate might also be more likely to have informed themselves enough to make the donation decision or are simply more likely to want to learn about the topic. As a methodological issue, using a survey to assess an individual's willingness to consent to the donation of a deceased relative's organs might not represent familial group-decision making processes that typically occur when making this decision. Finally, this study is limited by focusing on willingness to donate. Effective procurement rates are not solely determined by an individual's willingness to donate since cause of death determines if an individual is even eligible to donate organs.

## Conclusion

This study has attempted to examine the influence of institutional setting along with other determinants such as social interactions, political affiliation, socio-economic and demographic variables in determining the willingness of individuals to donate their own organs and those of their relatives. Our evidence draws from representative data from the EU about individuals' willingness to donate their own organs and those of a relative. We reach several key findings that support previous research on organ donation regarding the influence of the institutional setting (presumed consent matters), social interactions and political affiliation (donation is an act of social involvement) as well as a set of controls such as age and education having some effect on willingness to donate.

This study has developed new findings by not only examining the influence of institutional effects and social interactions on the willingness to donate but also by using interactive variables to investigate how type of donation legislation and awareness of this legislation together contribute to willingness to donate. This paper has demonstrated the influence of awareness of institutional setting on individuals being more willing to donate their organs or consent to the donation of their relative's organs and calls for greater attention to be paid to the role of institutional design in shaping individuals' attitudes about organ donation. The number of individuals respondents can count on and the easier it is to get help from a neighbor in case of serious problems appear to have a positive relationship with willingness to donate organs posthumously and to consent to the donation of a relative's. Against this argument, however, is a possible caveat in the potential endogeneity of social interactions [4] and institutional setting [31] in the long run, which calls for further analysis using longitudinal data. However, these findings seem robust given the controls introduced and tests run to check the model and seem to indicate that public policy plays a direct role though legislation and awareness campaigns about legislation type, and an indirect one by recognizing that social networks may shape the willingness of individuals to donate their organs.

## Competing interests

The author(s) declare that they have no competing interests.

## Authors' contributions

EM identified and acquired the data source. All authors performed statistical analysis of the data. All authors participated in the interpretation of data. All authors also participated in the drafting and revising of the manuscript. All authors read and approved the final manuscript.

## Pre-publication history

The pre-publication history for this paper can be accessed here:



## Supplementary Material

Additional file 1 Summary statistics. Summary statistics and variable definitions for all endogenous and explanatory variables included in analysis. Click here for file
