# Supplementary material for: Does organ donation legislation affect individuals' willingness to donate their own or their relative's organs? Evidence from European Union survey data
Source: BMC Health Serv Res. 2008 Feb 27;8:48. doi: 10.1186/1472-6963-8-48 (PMC2292708; doi:10.1186/1472-6963-8-48)
Supplement: Additional file 1 — Summary statistics. Summary statistics and variable definitions for all endogenous and explanatory variables included in analysis. [file 1472-6963-8-48-S1.doc]

**Additional file 1**

**Table A1. Summary statistics**

| **Variable** | **Define** | **Mean** | **Std. Err.** |
| --- | --- | --- | --- |
|  | **Endogenous variables** |  |  |
| Willingness to donate own organs | Willingness to donate own organs^a^ | 0.601 | 0.004 |
| Willingness to donate relative’s organs | Willingness to donate (consent) relative organs | 0.484 | 0.004 |
|  | **Explanatory variables** |  |  |
| *Needs and Related Socio-Demographics* | | | |
| Illness | Individuals suffers from a major illness | 0.272 | 0.003 |
| Very bad health | Very bad self-perceived health | 0.012 | 0.001 |
| Bad health | Bad self-perceived health | 0.051 | 0.020 |
| Good health | Good self-perceived health | 0.660 | 0.010 |
| Male | Individual is male | 0.462 | 0.004 |
| Age under 30 | Age less that 30 | 0.152 | 0.003 |
| Age 30 to 45 | Age 30-45 | 0.276 | 0.004 |
| Age 45 to 60 | Age 45-60 | 0.248 | 0.003 |
| Age over 60 | Age more than 60 | 0.324 | 0.004 |
| *Political Affiliation* | | | |
| Left politics | Self identified as left wing | 0.241 | 0.003 |
| Center politics | Self identified as center | 0.345 | 0.004 |
| Right politics | Self identified as right wing | 0.183 | 0.003 |
| Don’t know political identification | Don’t know political identification | 0.231 | 0.003 |
| *Social Interactions* | | | |
| Support from no one | In case of serious problems could count on no one | 0.036 | 0.001 |
| Support from 1 to 2 others | In case of serious problems could count on 1 or 2 | 0.341 | 0.004 |
| Support from 3 to 5 others | In case of serious problems could count on 3-5 | 0.350 | 0.004 |
| Support from over 5 others | In case of serious problems could count on +5 | 0.249 | 0.003 |
| Very easy to receive help from neighbors | Very easy practical help from neighbors | 0.268 | 0.003 |
| Easy to receive help from neighbors | Easy practical help from neighbors | 0.462 | 0.004 |
| Difficult to receive help from neighbors | Difficult practical help from neighbors | 0.063 | 0.002 |
| Very difficult to receive help from neighbors | Very difficult practical help from neighbors | 0.065 | 0.002 |
| *Socio-Economics* | | | |
| Rural | Individual lives in rural area | 0.370 | 0.004 |
| Stopped education at 15 years | Finished education at 15 or below | 0.259 | 0.003 |
| Stopped education at between 16-19 years | Finished education at 16-19 | 0.391 | 0.004 |
| Stopped education at over 20 years | Finished education at 20+ | 0.251 | 0.003 |
| Still studying | Still studying | 0.099 | 0.002 |
| *Institutional Setting and Knowledge* | | | |
| Awareness | Aware of the legislation on organ donation in its country | 0.310 | 0.003 |
| Presumed consent | Country has presumed consent | 0.319 | 0.004 |
| Presumed consent enforced | Country has presumed consent enforced | 0.287 | 0.004 |
| Informed consent | Country has informed consent | 0.390 | 0.004 |
| *Control Variables* | | | |
| Excellent cooperation | Excellent cooperation with interviewer | 0.597 | 0.004 |
| Fair cooperation | Fair cooperation with interviewer | 0.304 | 0.004 |
| Average cooperation | Average cooperation with interviewer | 0.087 | 0.002 |
| Bad cooperation | Bad cooperation with interviewer | 0.012 | 0.001 |
